# Strategic sequencing of orthodontic treatment and periodontal regenerative surgery: A literature review

**DOI:** 10.1016/j.jds.2025.03.010

**Published:** 2025-03-20

**Authors:** Kang-Wei Tu, Chia-Hwei Kuo, Chun-Cheng Hung, Da-You Yan, Jimmy Lian Ping Mau

**Affiliations:** a Department of Periodontics, Chi Mei Medical Center, Tainan, Taiwan; b Department of Orthodontics, Chi Mei Medical Center, Tainan, Taiwan; c School of Dentistry, College of Dental Medicine, Kaohsiung Medical University, Kaohsiung, Taiwan; d Division of Prosthodontics, Department of Dentistry, Kaohsiung Medical University Hospital, Kaohsiung, Taiwan; e Division of Periodontics, Department of Dentistry, Kaohsiung Medical University Hospital, Kaohsiung, Taiwan; f Yuya Dental Clinic, Tainan, Taiwan; g Department of Senior Welfare and Services, Southern Taiwan University of Science and Technology, Tainan, Taiwan

**Keywords:** Interdisciplinary approach, Orthodontic movements, Orthodontic treatment, Periodontal regenerative surgery

## Abstract

The interrelationship between orthodontic treatment and periodontal health is critical in managing patients with complex dental and periodontal needs. Orthodontic treatment offers functional and esthetic improvements but may also pose risks to periodontal health. For patients with periodontal disease requiring orthodontic intervention, thorough assessment and meticulous treatment planning are essential. Periodontal phase I therapy must precede orthodontic treatment to ensure periodontal stability. Once periodontal health is achieved, the sequence of orthodontic treatment and periodontal regenerative surgery becomes a pivotal decision. Performing regenerative surgery prior to orthodontic treatment can address bony defects that might otherwise hinder orthodontic progress and exacerbate bone loss under orthodontic forces. Alternatively, initiating orthodontic treatment before surgery can optimize defect morphology, reduce defect size, and enhance the outcomes of regenerative therapies. This literature review evaluates the impact of orthodontic treatment on periodontal bone defects and explores treatment sequencing principles. Key areas of discussion include the timing and prioritization of orthodontic alignment versus periodontal surgery and the effects of specific orthodontic movements—such as extrusion, intrusion, bodily movement, and uprighting—on periodontal defects. The choice of sequencing depends on factors such as defect type, disease severity, and individual patient considerations. This review underscores the importance of an interdisciplinary approach that integrates biomechanical principles and periodontal health to minimize risks and maximize regenerative potential. By strategically combining orthodontic and periodontal therapies, clinicians can achieve superior functional and esthetic outcomes in patients with challenging periodontal conditions.

## Introduction

The strategic sequencing of orthodontic treatment and periodontal regenerative surgery remains a critical focus of research in modern dental practice, particularly for patients with periodontally compromised dentition. While orthodontic treatment primarily aims to align teeth and correct malocclusions, it simultaneously exerts mechanical forces on the periodontal tissues,^1^^,^^2^ inducing alveolar bone remodeling and triggering physiological adaptation along with minor, reversible injury to the periodontium. Bone remodeling in response to orthodontic forces relies on the periodontal ligament (PDL), where applied forces influence both the speed and type of bone response. Light forces promote rapid osteoclast recruitment, whereas heavier forces may delay tooth movement due to blood vessel occlusion and hyalinization.^3^^,^^4^ Moreover, orthodontic therapy can significantly alter the oral microbial composition, often increasing periodontal pathogens, emphasizing the need for rigorous oral hygiene during treatment.^5^

These biomechanical and microbial changes can exacerbate pre-existing periodontal conditions.^6^ Periodontal regenerative surgery, by contrast, aims to restore lost periodontal structures, including bone and soft tissue, to enhance the stability and functionality of teeth. While orthodontic treatment and periodontal regeneration may have synergistic benefits, their treatment sequence significantly influences clinical outcomes. Improper sequencing may compromise healing, impede bone regeneration, or result in further periodontal destruction.

The interaction between orthodontic forces and periodontal tissues, particularly in patients with existing periodontal disease, underscores the need for carefully coordinated treatment planning.^7^, ^8^, ^9^ After successful non-surgical periodontal therapy, the treatment sequencing can be categorized into (1) Periodontal regenerative surgery first, followed by early orthodontic intervention (within 10 days postoperatively),^10^^,^^11^ (2) Periodontal regenerative surgery first, followed by late orthodontic intervention (at least 12 months postoperatively),^9^^,^^12^ and (3) Orthodontic treatment first, followed by periodontal surgery.

Traditionally, late orthodontic intervention following periodontal regenerative surgery has been considered a stable treatment sequence, with studies recommending a healing period of at least 12 months based on scientific evidence.^9^^,^^12^ However, this approach requires a prolonged treatment duration and may limit certain biological advantages associated with early tooth movement. Recent studies reveal that early orthodontic intervention may benefit a stable periodontal environment, facilitating more efficient tooth movement, enhancing bone regeneration, and potentially improving periodontal regenerative outcomes.^10^^,^^11^^,^^13^ The optimal timing of orthodontic intervention—whether early or late—remains a subject of debate.

Despite extensive research, non-surgical and regenerative periodontal therapy before orthodontic treatment is conservative and commonly recommended. However, emerging evidence suggests that under stable periodontal conditions, initiating orthodontic treatment first may not only enhance tooth movement but also improve bony defect morphology. The optimal treatment sequence remains uncertain, as various clinical factors—including patient health, periodontal stability, and treatment objectives—must be carefully evaluated to determine the most appropriate approach.^4^^,^^14^

Therefore, this literature review aims to critically analyze existing studies to explore the potential opportunities for alternative sequencing strategies, particularly the feasibility of periodontal regenerative surgery followed by early orthodontic intervention and orthodontic treatment prior to periodontal surgery. By establishing evidence-based strategies, this review seeks to provide clinicians with comprehensive guidelines for optimizing clinical outcomes in patients requiring both orthodontic and periodontal interventions.

## The effect of orthodontic movement on periodontal tissues

Orthodontic tooth movement involves alveolar bone remodeling in response to mechanical forces, combining physiological adaptation with minor, reversible injury to the periodontium.^1^ Bone remodeling is the balance between bone resorption in areas under pressure and bone formation in areas under tension, as teeth respond to applied biomechanical forces.^2^^,^^4^^,^^14^

The ability of teeth to move through the bone relies on the PDL.^3^ The pressure-tension theory explains that applied force compresses the PDL on one side and stretches it on the other, altering blood flow and triggering the release of molecular mediators such as prostaglandins and cytokines, including interleukin-1β. These mediators regulate cellular activities, promoting bone resorption on the compression side and bone formation on the tension side. Force magnitude influences these processes: light force maintains partial blood flow, enabling rapid osteoclast recruitment locally or via circulation, facilitating frontal resorption and initiating tooth movement within 2 days. Heavy force, in contrast, causes blood flow restriction and cell death (hyalinization) under compression, delaying movement until undermining resorption removes the lamina dura, typically requiring 7–14 days. Tooth movement involves a combination of frontal and undermining resorption, driven by a tightly regulated interplay of cellular and molecular responses.^4^^,^^14^

Orthodontic therapy induces rapid changes in oral microbial composition, accompanied by a decrease in saliva pH during the course of treatment,^6^ shifting from aerobic to anaerobic bacteria associated with active periodontitis. Within 12 days of treatment initiation, an increase in periodontal pathogenic bacteria such as spirochetes, fusiform bacteria, and Prevotella intermedia is observed. By 6 weeks, cocci levels decrease while spirochetes and motile rods increase, and by 3 months, bacteria from the red and orange complexes become established. These changes, often linked to decreased plaque control, result from the diminishing effectiveness of daily oral hygiene practices caused by fixed orthodontic appliances, particularly banding apparatus, associated with increased inflammation and loss of attachment.^15^^,^^16^ This highlights the importance of oral hygiene care during orthodontic treatment.

Research shows a slight worsening of periodontal status after orthodontic therapy.^17^ Tooth movement in patients with a reduced but healthy periodontium does not result in significant further loss of attachment.^18^ With meticulous orthodontic planning, routine follow-ups and enhanced oral hygiene maintenance, periodontal patients can successfully undergo orthodontic treatment following non-surgical periodontal therapy.

## The importance of non-surgical periodontal therapy before orthodontics

Periodontally compromised patients often present with orthodontic issues such as maxillary anterior tooth proclination, irregular interdental spacing, rotations, overeruption, migration, or tooth loss. These dental changes result from reduced periodontal support and can hinder periodontal treatment by complicating oral hygiene, impairing function, and compromising aesthetics.^19^

Orthodontic treatment for patients with periodontitis should follow a clear sequence, starting with non-surgical periodontal therapy. From a clinical viewpoint, erythematous and gingival swelling, increased periodontal probing depth (PPD), and bleeding on probing (BOP) are common characteristics of periodontal disease. An unstable periodontium, when subjected to orthodontic forces, will lead to more severe damage. Therefore, a comprehensive examination is essential.^4^^,^^14^

Firstly, we should focus on the patient's past medical history with concomitant medications, dental history, self-care practices, and smoking history. Secondly, a full-mouth periodontal data collection should be performed, including PPD, BOP, plaque index, clinical attachment level (CAL), furcation involvement, and tooth mobility. Additionally, the radiographic assessment provides information on the bone level. Finally, we should also document the intraoral photographs.^4^^,^^14^

Improvement in oral health through cause-related therapy should involve the removal of pathogenic factors via scaling, root planing, and patient education to reinforce oral hygiene and ensure compliance. Consequently, a re-evaluation should be performed. The treatment goals of non-surgical periodontal therapy should include: (1) full mouth bleeding score < 15 %, and (2) full mouth plaque score < 15 %.^20^ Additionally, favorable factors include high levels of patient compliance, absence of adverse habits (such as smoking), uncontrolled diabetes or other systemic diseases, and lower stress levels.^20^ Once the initial therapy has proven successful, as indicated by controlled inflammation and stable periodontal health, active orthodontic treatment can commence.^10^

## The sequencing of orthodontic treatment and periodontal regenerative surgery

The optimal sequencing for initiating orthodontic treatment and periodontal regenerative therapy remains a topic of considerable discussion. Treatment strategies can be categorized into two approaches: (1) periodontal regenerative surgery first, followed by orthodontic treatment, and (2) orthodontic treatment first, followed by regenerative surgery.

In the first approach, after non-surgical and regenerative periodontal treatments, this can create favorable pre-orthodontic conditions. When periodontal surgery is performed first, the approach can be further subdivided into early orthodontic intervention (within 10 days postoperatively)^10^^,^^11^ and late orthodontic intervention (at least 12 months postoperatively).^9^^,^^12^

Late orthodontic intervention has been considered a more conservative approach, ensuring stable regenerative outcomes before initiating orthodontic movement.^7^^,^^8^ Prato et al.^10^ proposed a decision-making algorithm to assist clinicians in determining the optimal timing of orthodontic intervention. According to their recommendations, orthodontic treatment should commence 6–9 months after open-flap debridement and at least 1 year after periodontal regenerative surgery. These timeframes are based on scientific evidence and wound healing principles.

Recently, several studies suggest that early orthodontic intervention (within 10 days postoperatively)^10^^,^^11^ may offer significant advantages, particularly in reducing overall treatment duration and enhancing periodontal healing outcomes.^21^, ^22^, ^23^, ^24^ Immediate tooth movement has not been shown to adversely affect periodontal regeneration; instead, it may facilitate favorable bone morphology by optimizing tooth alignment, inhibiting epithelial apical down-growth, and promoting new attachment formation.^10^^,^^13^ Additionally, early intervention takes advantage of the regional acceleratory phenomenon induced by periodontal surgery, which temporarily enhances bone turnover and remodeling, potentially accelerating orthodontic movement.^25^

Dung et al.^11^ investigated the long-term outcomes of early orthodontic intervention following periodontal regenerative surgery. After a mean follow-up of 12.8 years, all treated teeth remained functional with no tooth loss. Clinical assessments demonstrated a significant reduction in PPD (mean: −3.94 mm) and an increase in CAL (mean: +3.47 mm). These findings suggest that early orthodontic intervention following periodontal regenerative therapy does not compromise periodontal healing and may lead to stable long-term outcomes.

The second approach involves initiating orthodontic treatment prior to regenerative surgery. This strategy has been shown to facilitate the reduction of periodontal defects and promote an increase in clinical attachment due to the repositioning of teeth within the alveolar bone. Furthermore, orthodontic forces can enhance cellular activity, acting as a stimulating factor for bone apposition, which may further support the regenerative process and improve overall periodontal health.^19^^,^^26^

In conclusion, both approaches—starting with periodontal regenerative surgery or initiating orthodontic treatment first—have their distinct advantages. The choice between these approaches depends on the clinical scenario, the extent of periodontal involvement, and the individual patient's needs.

## Different orthodontic movements in periodontal defects

In periodontally compromised teeth, the center of resistance shifts apically due to changes in the periodontium, resulting in greater moments during force application and an enhanced extrusion component in the applied force.^27^ In uncontrolled periodontitis, orthodontic movement of teeth into infrabony pockets can damage periodontal attachment, particularly when correcting teeth that have become tipped or elongated due to periodontal disease.^28^ However, orthodontic treatment is not always contraindicated for adults with severe periodontal conditions. As long as periodontitis is controlled, adjuvant orthodontic therapy is often essential for enhancing the restoration of a compromised dentition.^19^ Common orthodontic movements including extrusion, intrusion, bodily movement, tipping, and uprighting have been reported as adjunctive therapies in the interdisciplinary management of infrabony defects.^10^

## Extrusion

Tooth extrusion, or orthodontic coronal displacement, mimics the natural eruption while using extrusive forces to extend beyond the limits. Extrusive displacement of non-restorable teeth can help gain vertical bone, as the bone regenerates through orthodontic extrusion. This approach may eliminate the need for a bone graft, facilitating implant placement in areas with insufficient bone.^29^^,^^30^

Experimental and clinical studies indicate that orthodontic extrusion of teeth with one- or two-wall infrabony defects improves connective tissue attachment and reduces defect size. This approach is particularly beneficial when osseous resection risks furcation exposure, endangers adjacent teeth, or involves maxillary sinus proximity, creating favorable conditions for subsequent surgical periodontal treatment.^31^ Ogihara and Wang's research^32^ focused on limited orthodontics combined with enamel matrix derivative (EMD) and demineralized freeze-dried bone allograft (DFDBA) versus EMD/DFDBA alone in treating two- or three-wall infrabony defects. The study found that while both approaches were effective, the addition of limited extrusive orthodontic forces showed greater benefits in improving attachment levels in two-wall defects.

Arsić et al.^33^ examined the effects of orthodontic extrusion and discovered that orthodontic extrusion increases the keratinized gingival zone and shifts the mucogingival junction, usually in the direction of tooth movement. Da Silva et al.^34^ demonstrated in a dog study that combining fiberotomy and scaling with orthodontic extrusion effectively prevents coronal tissue displacement.

In general, orthodontic extrusion relocates plaque coronally, making it safe for periodontium. It can also promote the regeneration of hard and soft tissues, enhancing conditions for subsequent surgical periodontal treatment. However, some studies recommend waiting a few months to allow inflammation to fully subside before initiating the procedure.^30^ Moreover, endodontic and prosthetic considerations are essential, as extruded teeth require gradual shortening for proper function and alignment.^10^

## Intrusion

Intrusion is a common approach for managing pathological tooth migration associated with periodontal disease.^24^^,^^35^ Orthodontic intrusion realigns migrated teeth to enhance aesthetics and function following periodontal therapy. This approach improves periodontal parameters such as clinical crown length and marginal bone levels. Additionally, orthodontic intrusion is also used to harmonize gingival margins, especially in protruded or malpositioned teeth, often in conjunction with restorative procedures, causing apical movement of the gingival margin.

The intrusion of teeth with a healthy but reduced periodontium reshapes the alveolar process without altering the extent of periodontal support. However, meticulous control of light orthodontic forces and infection management are essential.^30^ Cardaropoli et al.^36^ demonstrated that intrusion was performed 7–10 days after periodontal surgery significantly reduced angular infrabony defects in migrated maxillary incisors.

Pinho et al.^37^ reported a case of anterior tooth intrusion using a miniscrew, demonstrating enhanced bone apposition in infrabony defects. A controlled clinical trial by Ghouraba et al.^38^ compared two treatment sequences for over-erupted teeth with angular bone loss: guided tissue regeneration before or after orthodontic intrusion. While guided tissue regeneration first provided short-term benefits, initiating treatment with orthodontic intrusion resulted in superior long-term improvements in pocket depth, tooth mobility, and defect resolution. The study suggests that orthodontic intrusion before guided tissue regeneration leads to more stable periodontal outcomes.

However, intrusion leads to potential adverse effects increasing the risk of further attachment loss by pushing marginal plaque deeper into the pocket,^2^ especially in case with narrow vertical bony defects. Antonarakis et al.^30^ suggest that, in a healthy but reduced periodontium, applying light forces (5–15 cN per tooth) can reshape the alveolar process without causing periodontal breakdown and may even enhance connective tissue attachment. Furthermore, they proposed that in cases of wide bony defects, orthodontic intrusion can be utilized first to enhance defect morphology before regenerative procedures. However, due to the risks of tooth intrusion, in cases of narrow bony defects, regenerative periodontal procedures should precede orthodontic intrusion.

## Bodily tooth movement

Periodontal bone loss shifts the center of resistance apically in the affected teeth, making bodily movement mechanics more challenging.^10^ Bodily tooth movement into infrabony defects can contribute to restoring a healthy, functional dentition with favorable outcomes.

Geraci's research^41^ on Rhesus monkeys induced artificial two- and three-wall osseous defects, demonstrating that bodily tooth movement into these defects facilitated full bone regeneration and healing. However, when performed in the presence of periodontal inflammation, it led to further periodontal breakdown.^5^ Similarly, a beagle dog study found that bodily tooth movement during orthodontic therapy may accelerate connective tissue attachment loss, particularly in inflamed infrabony pockets, increasing the risk of further attachment loss when the tooth is moved into the defect.^28^ Nonetheless, it is important to recognize that bony defect repair in animal models may be largely influenced by species-specific bone metabolism, which differs significantly from human alveolar bone dynamics.

In the human research, when moving through cortical bone, the process may be hindered, potentially leading to buccal and lingual bone dehiscences. In such cases, periodontal regenerative surgery or even bone augmentation to increase alveolar bone width is recommended before initiating orthodontic movement.^39^ Cardaropoli et al.^40^ demonstrates the efficacy of an approach in which periodontal regenerative surgery first, followed by early orthodontic intervention in treating infrabony defects, achieving PPD reduction, and CAL gain.

## Tipping

Tipping occurs when a single force is applied to the crown of a tooth, causing the tooth to rotate around its center of resistance, located roughly halfway along the root. As the tooth rotates, the PDL experiences compression—near the root apex on the same side as the spring and at the alveolar crest on the opposite side.^4^ Orthodontic tipping movement promotes bone remodeling, enhances healing in periodontal defects, and reduces angular infrabony defects, although it does not result in connective tissue attachment gain, with varying effects on bone apposition and junctional epithelium.^42^

Vardimon et al.^43^ studied mesial tipping of maxillary first molars in 52 Wistar rats with alveolar defects, finding that orthodontic movement occurred only in the treated group, where bone apposition was 6.5 times greater than in controls, demonstrating its role in bone remodeling. Cirelli et al.^44^ studied four dogs and found that tipping movements toward one-wall intraosseous defects post-surgery did not hinder healing but slightly reduced linear bone apposition. Nemcovsky et al.^45^ revealed that tipping of the first molars mesially towards the defects showed that orthodontic tooth movement may reduce angular infrabony defects, enhance connective tissue healing, and limit junctional epithelium down-growth, with healing involving long junctional epithelium.

## Uprighting

Molar uprighting is achieved through pure rotational forces using a high moment-to-force ratio, ensuring the center of rotation aligns near the center of resistance.^46^ Tilting tooth happens when its adjacent tooth is missing for an extended period, often leading to periodontal issues on the mesial side of the tilted tooth, mostly shown in the molar region. Molar uprighting through orthodontic movements can correct the supporting tissue defects associated with a tipped molar, facilitating predictable and standard periodontal surgical procedures. Addressing the osseous defect surgically without preceding orthodontic treatment would necessitate removing an excessive amount of bone. In the presence of periodontal pockets, orthodontic uprighting movements can widen the mesial angular defect and reduce the pocket depth. Additionally, the attached gingiva will also adopt a more natural contour, the axial alignment significantly improves, and the occlusion becomes more functional and satisfactory.^47^^,^^48^

Mandibular second molar impactions may also cause distal bony defects of the first molars. By uprighting impacted mandibular second molars, infrabony periodontal defects on the distal surfaces of the first molars improved, as the alveolar bone was elevated coronally, supporting the long-term periodontal health.^49^ Many clinicians recommend using an uprighting spring combined with a stabilizing archwire that passes through the molar tube, applying an intrusive force to the molar that is equal and opposite to the force exerted by the uprighting spring.^50^

## Clinical prioritization of treatment sequencing

The interaction between these treatments requires careful consideration, especially when facing infrabony defects under stable periodontal conditions, as improper sequencing may disrupt healing and compromise treatment outcomes. When considering the sequencing of orthodontic and periodontal regenerative treatments, two main strategies have emerged.

## Periodontal regenerative surgery first

In this approach, intrusion into narrow defects and bodily movement are orthodontic movements that should be performed after periodontal regenerative surgery.

Intrusion is often used to manage pathological extrusion and align teeth with a healthy but reduced periodontium,^24^^,^^30^^,^^35^ and increasing the risk to pushing marginal plaque into deep and narrow pockets.^2^^,^^30^ Similarly, bodily tooth movement into infrabony defects can induce inflammation leading to further periodontal breakdown. Bodily movement should be done after the periodontium has been stabilized through regenerative procedures to avoid periodontium damage.^5^^,^^10^^,^^28^

Recent studies suggest that early orthodontic intervention, performed within 10 days after regenerative surgery, may facilitate new attachment formation without negatively impacting healing. This early intervention has been shown to enhance the regenerative process by promoting favorable changes in the PDL and bone remodeling.^10^^,^^13^ On the other hand, late orthodontic intervention, performed at least 12 months after periodontal regeneration, allows for stable and predictable periodontal outcomes, as the tissues have had more time to heal and stabilize.^9^^,^^12^

## Orthodontic treatment first

Alternatively, extrusion, intrusion into wide defects, tipping and uprighting are orthodontic movements that can be initiated before periodontal regenerative surgery.

Extrusion can create vertical bone gain through orthodontic tooth movement, mimicking the natural direction of physiological eruption, thus eliminating the need for a bone graft in certain cases.^29^^,^^30^ Intrusion into wide defects allows for easier plaque control than in narrow defects, and initiating orthodontic treatment first can create a more favorable defect morphology for periodontal regeneration.^30^

Tipping movement promotes bone remodeling, enhances healing in alveolar and periodontal defects, and reduces angular infrabony defects. It typically results in varying effects on bone apposition and junctional epithelium, but without connective tissue attachment gain.^42^^,^^43^^,^^45^ Molar uprighting, particularly for teeth tipped due to tooth loss or impaction, can facilitate periodontal regeneration by improving the alignment of the teeth and enabling more predictable surgical outcomes. These movements can also promote better tooth positioning within the alveolar bone, which enhances bone regeneration and periodontal health. However, it is crucial to carefully manage the forces applied during these orthodontic movements to prevent exacerbating periodontal conditions.^47^^,^^49^^,^^50^

## Conclusion

This literature review emphasizes the importance of a comprehensive periodontal evaluation and the initial stabilization through non-surgical periodontal therapy. The strategic sequencing of orthodontic treatment and periodontal regenerative surgery is essential for achieving optimal outcomes in patients with periodontally compromised dentition.

Periodontal regenerative therapy should precede orthodontic treatment for intrusion into narrow defects and bodily movement, whether through early or late orthodontic intervention. Both strategies show promising results, yet further research is required to elucidate their long-term effects and determine the ideal timing. Conversely, orthodontic treatment is prioritized for extrusion, intrusion into wide defects, tipping, and uprighting movements.

Ultimately, individualized treatment planning remains the cornerstone of achieving predictable and stable clinical outcomes. Further advancing research in this field will help establish precise guidelines for integrating orthodontic and periodontal regenerative therapies, optimizing outcomes while supporting evidence-based decision-making in clinical practice.

## Declaration of competing interest

The authors have no conflicts of interest relevant to this article.
